# The Prevalence and Related Risk Factors of Urinary Incontinence Among Adult Women in Al Medina Al Munawara, Saudi Arabia

**DOI:** 10.7759/cureus.64966

**Published:** 2024-07-20

**Authors:** Mohammad F Alonezy, Ahmed S Metwally, Osama A Alhazmi, Albaraa O Alrehaili, Abdullah A Almohammadi, Abdulaziz S Aljuhani, Faisal M Alharthi, Nawaf A Aloufi

**Affiliations:** 1 Family and Community Medicine, Taibah University, Medina, SAU; 2 Family Medicine, Suez Canal University, Ismailia, EGY

**Keywords:** urinary incontinence, adult females, kingdom of saudi arabia (ksa), al medina al munawara, risk factors, prevalence

## Abstract

Introduction

Urinary incontinence (UI) is a common clinical problem. It has an impact on an individual's social, professional, psychological, and physical elements of life. The present study aimed to identify the prevalence of UI and associated risk factors among Saudi women in Al Medina Al Munawara, Saudi Arabia.

Methods

A cross-sectional study was conducted using an online questionnaire among 430 women aged 18-60 in Al Medina Al Munawara. The participants completed the validated Arabic version of the International Consultation on Incontinence Questionnaire Short Form (ICIQ-SF) to assess the prevalence of UI and its associated factors.

Results

Among the participants, 64.8% did not experience urine incontinence, while 17.8% reported slight, 14.0% reported moderate, and 3.3% reported severe incontinence. Stress incontinence caused by coughing or sneezing was the most common cause (48.6%), followed by before reaching the toilet (urge incontinence) (34.5%) and after urination (15.5%). Significant associations were found between age, marital status, number of children, diabetes mellitus, urinary tract diseases, previous abdominal or pelvic surgery, obesity, constipation, and menopausal symptoms. However, no significant association was found between pregnancy and urine incontinence.

Conclusions

This study reveals a moderate prevalence of UI among Saudi women in Al Medina Al Munawara. The findings highlight the importance of early detection, treatment, and education on pelvic floor exercises to address UI. Factors such as age, marital status, number of children, and various medical conditions are associated with this condition, emphasizing the need for comprehensive management strategies.

## Introduction

Urinary incontinence (UI) is a common clinical problem, particularly among women [1]. The International Urogynecological Association (IUGA)/International Continence Society (ICS) defines it as a complaint of involuntary urine pass [2]. UI is divided into several categories: first, stress incontinence, which is an uncontrollable flow of urine after physical effort or movements like coughing, sneezing, or laughing; second, urgency incontinence, which is described as an uncontrollable need to urinate that strikes suddenly and without warning, in which the unintended urine leak occurs concurrently; third, mix incontinence, which combines urine incontinence brought on by stress and urgent conditions [2].

UI is a prevalent, unpleasant condition that primarily affects women. When it is not, it may be wrongly believed to be a typical aspect of aging that does not require medical attention and can even be disregarded. Contrary to popular belief, which holds that it is more prevalent in the older population, it is seen in women of all ages and from many cultures and ethnicities, making it a global issue [3]. UI appears to be underdiagnosed and underreported because many patients do not report the disease to their physician for two reasons: the first is their perception that UI is a normal result of age that cannot be cured, and the second is their fear of embarrassment [4].

UI affects around 423 million individuals worldwide, with women being three times more likely than males [5]. UI affects almost one-third of women over the age of 40 [6]. The stated prevalence of UI in the literature ranges from 5% to 70% globally [4]. According to a systematic literature analysis comprising research from 12 countries, UI has a broad prevalence range ranging from 16.2% to 81.9% [3]. The total prevalence of urine incontinence in the Middle East ranges from 20.3% to 54.8% [7]. In Saudi Arabia, the prevalence of UI ranged from 29% to 56% [8]. Urinary continence was reported to be 41.4% and 29% in the cities of Jeddah and Riyadh [9].

UI relates to several risk factors, including age, obesity, medical comorbidities, hysterectomy, and multiparity. Furthermore, recurrent pregnancies and deliveries may be a significant risk factor for young and middle-aged women [10]. As a result, identifying the risk factors is critical in preventing UI, which will assist in reducing the negative impact of incontinence on quality of life and social activities that are frequent among women with incontinence [3,11].

The present study aimed to identify the prevalence of UI and associated risk factors among Saudi women in Al Medina Al Munawara, Saudi Arabia by questionnaire.

## Materials and methods

Study design

A cross-sectional study was conducted using a population-based online questionnaire. The questionnaire is published online for the adult women's community in Al Medina, Saudi Arabia. The population of the women's community in the Al Medina region is close to 900,000 people. The study included all patients aged 18-60 years, with or without comorbidities. Cases who refused to participate in the study and cases below the age of 18 years old and above the age of 60 years old were excluded from the study.

Sample size

The study's sampling unit was the adult female community in Al Medina, Saudi Arabia, including Saudi and non-Saudi women. We calculated the sample size by using the OpenEpi.com website, assuming that the anticipated frequency is 50%, the confidence level is 95%, and the confidence limit is 5+ with a total number of 384 participants at least needed. A total of 430 participants completed the questionnaire.

Sampling technique and method

A self-declarative electronic questionnaire form (Google Forms) was used to assess the prevalence of UI and associated factors among adult females in Medina, Saudi Arabia using convenience sampling. The questionnaire is made up of the preparatory phase and implementation phase. The preparatory phase consists of two sections. The first section is about related factors that can affect UI in females, such as age, ethnicity, marital status, parity, and other comorbidities. In the second section, we used the validated Arabic version of the International Consultation on Incontinence Questionnaire Short Form (ICIQ-SF) [12]. In the implementation phase, the questionnaire was distributed online through social media applications (WhatsApp, Twitter, and Telegram) for the women's community in Medina, Saudi Arabia. After an explanation of the study's purpose, all eligible women were asked to fill out the questionnaire. Data were collected over a period of 12 weeks. 

Data management and analysis plan

Data was managed and analyzed using Statistical Package for Social Sciences (SPSS) version 25 (IBM Corp., Armonk, NY, USA); the p-value was considered statistically significant when it is less than 0.05. For continuous variables, mean and standard deviation were analyzed using a student's t-test or ANOVA test for categorical data, and a chi-square test was used to assess differences in proportions across the categories.

Ethical considerations

Approval of study protocol and data collection was obtained from the Research Ethical Committee at Taibah University, Madinah. The objectives of the study were explained to the participants, and informed consent was obtained from each participant. The collected data will be confidential and will not be disclosed except for study purposes.

## Results

A total of 430 women initially participated in the study. Nine participants did not meet the criteria and were excluded from the analysis. Thus, data from 421 individuals who met the inclusion criteria and completed the survey were included in the analysis. The age distribution of the participants revealed that the majority fell into the 18-29 age group (248 (58.9%)), followed by the 30-39 age group (53 (12.6%)), 40-49 age group (79 (18.8%)), and 50-59 age group (41 (9.7%)). In terms of marital status, most participants reported being single (242 (57.5%)), while 165 (39.2%) were married, and a small percentage were divorced (14 (3.3%)). The study population was predominantly comprised of Saudi nationals (411 (97.6%)), with a minority being non-Saudi (10 (2.4%)). Regarding the participants' health status, the majority (269 (63.9%)) reported no previous vaginal deliveries. A smaller proportion reported one to two vaginal deliveries (45 (10.7%)), three to four vaginal deliveries (60 (14.3%)), and more than four vaginal deliveries (47 (11.2%)). For medical history, a small percentage of participants reported having diabetes mellitus (23 (5.5%)) and hypertension (23 (5.5%)). Additionally, a percentage of participants reported a history of urinary tract diseases (31 (7.4%)), while 91 (21.6%) reported having undergone previous abdominal or pelvic surgery (Table 1)

**Table 1:** Demographic characteristics and medical history of the study sample

Variable	Parameters	Frequency (n=421)	(%)
Age group	18-29 years	248	58.9
	30-39 years	53	12.6
	40-49 years	79	18.8
	50-59 years	41	9.7
Marital status	Single	242	57.5
	Married	165	39.2
	Divorce	14	3.3
Nationality	Saudi	411	97.6
	Non-Saudi	10	2.4
Education	Secondary or below	96	22.8
	University	303	72.0
	Master's degree or above	22	5.2
Number of children	No children	244	58.0
	1-3	65	15.4
	4-6	92	21.9
	More than 6	20	4.8
Number of abortions	None	327	77.7
	1-2	74	17.6
	3 or more	20	4.8
Number of vaginal deliveries	None	269	63.9
	1-2	45	10.7
	3-4	60	14.3
	More than 4	47	11.2
Medical history	Diabetes mellitus	23	5.5
	Hypertension	23	5.5
	Urinary tract diseases	31	7.4
	Previous abdominal or pelvic surgery	91	21.6

Table 2 presents additional characteristics reported by the participants. Obesity was observed in 79 (18.8%) of the sample. Constipation and menopausal symptoms were reported by 75 (17.8%) and 49 (11.6%) of the participants, respectively. Caffeine use was prevalent, with 329 (78.1%) of the participants reporting its consumption. Soft drink consumption was reported by 150 (35.6%) of the participants. Smoking was observed in 20 (4.8%) of the participants.

**Table 2:** Distribution of other characteristics of the study sample

Variable	Frequency (n=421)	(%)
Obesity	79	18.8
Constipation	75	17.8
Menopausal symptoms	49	11.6
Diuretics use	16	3.8
Caffeine use	329	78.1
Soft drink use	150	35.6
Smoking	20	4.8

**Figure 1:**
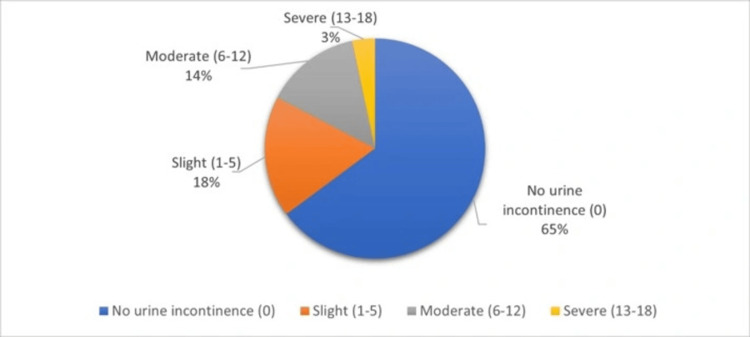
Distribution of ICIQ-UI SF score among the study sample ICIQ-UI SF, International Consultation on Incontinence Questionnaire - Urinary Incontinence Short Form

Figure 1 displays the scores on the International Consultation on Incontinence Questionnaire - Urinary Incontinence Short Form (ICIQ-UI SF) among the study sample. Most participants (273 (64.8%)) reported no urine incontinence, as indicated by a score of zero on the ICIQ-UI SF. A proportion of participants (75 (17.8%)) were classed as having slight urine incontinence, with scores ranging from one to five. Moderate urine incontinence, with scores ranging from six to 12, was observed in 59 (14.0%) of the participants. A smaller percentage of participants (14 (3.3%)) experience severe urine incontinence, with scores ranging from 13 to 18.

**Figure 2:**
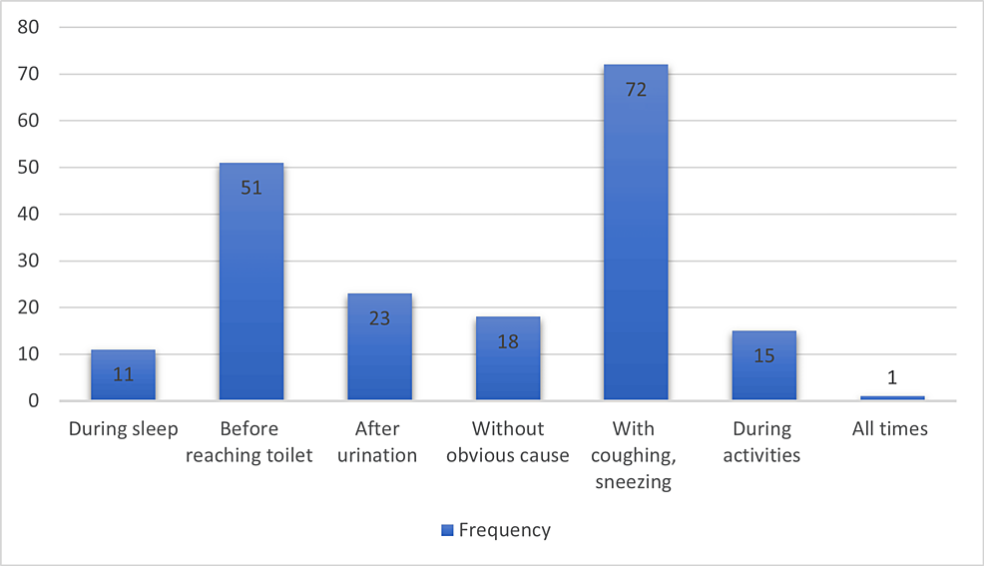
Cause of urine incontinence among cases

Figure 2 presents the causes of urine incontinence among the identified cases within the study sample. A total of 148 participants were considered cases for the analysis. Among the cases, the most common cause of urine incontinence was reported as "with coughing, sneezing" (72 (48.6%)). This was followed by "before reaching the toilet" (51 (34.5%)) and "after urination" (23 (15.5%)). A smaller percentage of cases reported urine incontinence during sleep (11 (7.4%)), without an obvious cause (18 (12.2%)), during activities (15 (10.1%)), and always (1 (0.7%)).

The association between urine incontinence and various sample characteristics was examined, and the results are presented in Table 3. The analysis included 273 participants without urine incontinence and 148 participants with urine incontinence. Significant differences were observed in the distribution of age between participants with and without urine incontinence (p<0.001). In the age group of 40 or more, 64 (53.3%) had urine incontinence, compared to 56 (46.7%) without urine incontinence. Moreover, a significant association was found between marital status and urine incontinence (p<0.001). Among married participants, 83 (56.1%) had urine incontinence, compared to 82 (30.0%) without urine incontinence. The number of children showed a significant association with urine incontinence (p<0.001). Among participants with no children, 185 (67.8%) had no urine incontinence. However, among those with no children, 59 (40.1%) reported urine incontinence. Regarding pregnancy, there was no significant association between pregnancy and urine incontinence (p=0.893). Moreover, number of vaginal deliveries was significantly associated with urine incontinence (p<0.001). Among participants with no vaginal deliveries, 196 (71.8%) had no urine incontinence. However, among those with one to two vaginal deliveries, 73 (49.3%) reported urine incontinence. Several other variables showed significant associations with urine incontinence. These include a history of diabetes mellitus (p=0.002), urinary tract diseases (p= 0.002), previous abdominal or pelvic surgery (p<0.001), obesity (p=0.001), constipation (p=0.010), and menopausal symptoms (p=0.031). No significant associations were found between urine incontinence and variables such as caffeine use, soft drink consumption, diuretic use, and smoking.

**Table 3:** Association between urine incontinence and sample characteristics We calculate the P-value by using the Chi-squared test

Variable	Parameters	No urine incontinence (n=273)	Urine incontinence (n=148)	P-value
Age	Less than 40	217 (72.1%)	84 (27.9%)	0.000*
	40 or more	56 (46.7%)	64 (53.3%)	
Marital status	Single	181 (66.3%)	61 (41.2%)	0.000*
	Married	82 (30.0%)	83 (56.1%)	
	Divorce	10 (3.7%)	4 (2.7%)	
Number of children	No children	185 (67.8%)	59 (40.1%)	0.000*
	1-3	34 (12.5%)	31 (21.1%)	
	4-6	47 (17.2%)	45 (30.6%)	
	>6	7 (2.6%)	12 (8.2%)	
Pregnancy	Yes	8 (2.9%)	4 (2.7%)	0.893
	No	265 (97.1%)	144 (97.3%)	
Number of vaginal deliveries	0	196 (71.8%)	73 (49.3%)	0.000*
	1-2	26 (9.5%)	19 (12.8%)	
	3-4	28 (10.3%)	32 (21.6%)	
	>4	23 (8.4%)	24 (16.2%)	
Number of abortions	0	233 (85.3%)	94 (63.5%)	0.000*
	1-2	32 (11.7%)	42 (28.4%)	
	3 or more	8 (2.9%)	12 (8.1%)	
Diabetes mellitus	Yes	8 (2.9%)	15 (10.1%)	0.002*
	No	265 (97.1%)	133 (89.9%)	
Hypertension	Yes	13 (4.8%)	10 (6.8%)	0.390
	No	260 (95.2%)	138 (93.2%)	
Urinary tract diseases	Yes	12 (4.4%)	19 (12.8%)	0.002*
	No	261 (95.6%)	129 (87.2%)	
Previous abdominal or pelvic surgery	Yes	43 (15.8%)	48 (32.4%)	0.000*
	No	230 (84.2%)	100 (67.6%)	
Caffeine	Yes	209 (76.6%)	120 (81.1%)	0.283
	No	64 (23.4%)	28 (18.9%)	
Soft drink	Yes	97 (35.5%)	53 (35.8%)	0.954
	No	176 (64.5%)	95 (64.2%)	
Obesity	Yes	39 (14.3%)	40 (27.0%)	0.001*
	No	234 (85.7%)	108 (73.0%)	
Constipation	Yes	39 (14.3%)	36 (24.3%)	0.010*
	No	234 (85.7%)	112 (75.7%)	
Menopausal symptoms	Yes	25 (9.2%)	24 (16.2%)	0.031*
	No	248 (90.8%)	124 (83.8%)	
Diuretics	Yes	7 (2.6%)	9 (6.1%)	0.072
	No	266 (97.4%)	139 (93.9%)	
Smoking	Yes	13 (4.8%)	7 (4.7%)	0.988
	No	260 (95.2%)	141 (95.3%)	

## Discussion

The main goal of this study was to assess the prevalence of female UI in Al-Medina, Saudi Arabia. The study findings reveal that among the 421 females surveyed, the prevalence of UI was 148 (35.82%) with 75 (17.8%) of them having slight UI, 59 (14.0%) having moderate UI, and 14 (3.3%) having severe UI. The prevalence of UI was lower in a similar study conducted in Riyadh (42.6%) of which 18.6% reported mild impact, 6.9% moderate, and 2.8% had severe impact [8]. In Jeddah (41.4%), 29.9% of them had limitations in their social activities [9]. While prevalence in a similar study in Taif was 16.8%, 64.3% of them had daily urinary leakage more than once [13]. In addition, Alghamdi et al. found in their study that among the participants, 454 (56.6%) of them reported having at least one UI [14]. The prevalence of UI was lower than in a study in Egypt (55%) with 65.3% of them having mild severity, 19.4% having moderate, and 15.3% had severe UI, while the prevalence was higher than in a study in Qatar (20.6%) [7,15]. In line with these findings, a study in Germany reported the prevalence of UI to be 48.3% among the women who participated in the study [16]. This shows that there is a slight variation in the prevalence of UI across areas and the regions in which the study was conducted.

The results reveal that the most common disposing factors of UI were coughing and sneezing (72 (48.6%)), followed by "before reaching the toilet" (51 (34.5%)), "after urination" (23 (15.5%)) followed by without an apparent cause (18 (12.2%)), and during activities (15 (10.1%)). Other less prevalent were “during the sleep” (11 (7.4%)) and “at all times” (1 (0.7%)). According to the study by Khowailed et al., most of the respondents experienced UI during exercises (64.2%) [17]. In comparison with a study by Alshenqeti et al., in King Khalid Hospital, the most common disposing factors of UI were coughing or sneezing (37.2%) or before reaching the bathroom (36.7%) [18].

The study also revealed a statistically significance difference across each risk factor: age, marital status, number of children, and vaginal deliveries among the individuals with and without UI (p<0.05). Notable variations were noted in the distribution of age, marital status, number of children, and vaginal deliveries. Similarly, a Riyadh-based study by Abduldaiem et al. established that the occurrence of UI is significantly influenced by age, multiparty, obesity, and hypertension risk factors [19]. In a study by Aly et al., in Egypt, statistically significant correlations between UI and laxative use, multi-parity, aging, osteoarthritis, stroke, and vaginal prolapse were found [20]. This implies that parity and age are among the major contributing factors for UI among women.

The findings of the study reveal a notable disparity in the prevalence of UI between participants aged 40 years and above compared to those below 40 years. Specifically, the percentage of individuals experiencing UI was markedly higher among the older age group, with 53.3% of participants aged 40 years and above reporting UI compared to only 27.9% among their younger counterparts. In Taif City in Saudi, a substantial increase in the risk of developing UI was seen in those 50 years of age or older with DM type 2, neuropathy, ovarian cyst, UTI, and elevated levels of FBG and HbA1c [21].

This study had both strengths and limitations. One of the primary strengths is the detailed assessment of various disposing factors, such as coughing, sneezing, and physical activities, which provides a comprehensive understanding of the triggers of UI in the population. Additionally, the study's comparison with similar studies conducted in different regions, including Riyadh and Taif, offers valuable comparisons in regional variations in the prevalence and factors associated with UI. Even with this, the study had a few limitations, the first being that the study was a cross-sectional analysis and, hence, had no follow-up, which prevented the observation of changes or trends in UI over time. Second, the reliance on self-reported questionnaires rather than clinical gynecological examinations, urodynamic studies, or other diagnostic tests may result in inaccuracies or underreporting of UI. Additionally, the study's focus on a single region, Al Medina, restricts the generalizability of the findings to the broader population of Saudi Arabia. Furthermore, the exclusion of individuals aged 60 years and older limits the comprehensiveness of the study, as this age group may exhibit different patterns or higher prevalence rates of UI. The sample size of 421 women, while sufficient for initial observations, might still need to be more significant to capture all the nuances and variability present in the population. 

## Conclusions

This study shows that the Al Medina region in Saudi Arabia has a moderate prevalence of female UI. The study also noted that UI is significantly influenced by age, marital status, number of children, menopausal symptoms, obesity, prior abdominal or pelvic surgery, diabetes mellitus, urinary tract diseases, and obesity as risk factors. Most of the women who have had this condition were affected differently depending on the level of severity of the condition. Therefore, this study acts as a critical first step toward bringing attention to the need for UI treatment and early detection in the Al Medina region of Saudi Arabia. This study may help inform healthcare systems that treat UI in women and may also aid in educating patients and medical professionals about the importance of early detection and treatment. Along with raising public awareness of pelvic floor exercises to strengthen the pelvic floor muscles, especially during and after pregnancy, primary prevention of UI and giving women with UI the necessary information and education should also be promoted. It would be crucial to evaluate the attitudes, knowledge, and convictions of medical personnel about UI. More research is advised in this area to evaluate the attitudes and knowledge of primary care physicians, as this will directly affect the care given to women. Women who experience incontinence symptoms may seek and receive care for their symptoms more quickly if the general public and medical professionals are educated about the factors that influence treatment seeking and the options available for treatment.
